# Ghrelin Modulates Lateral Amygdala Neuronal Firing and Blocks Acquisition for Conditioned Taste Aversion

**DOI:** 10.1371/journal.pone.0065422

**Published:** 2013-06-07

**Authors:** Lige Song, Qianqian Zhu, Tianwei Liu, Ming Yu, Kewei Xiao, Qingnuan Kong, Renliang Zhao, Guo-Dong Li, Yu Zhou

**Affiliations:** 1 Department of Physiology, Medical College of Qingdao University, Qingdao, Shandong, China; 2 Department of Neurology, Medical College Affiliated General Hospital, Qingdao, Shandong, China; 3 Research Institute of Cerebrovascular Diseases, Medical College of Qingdao University, Qingdao, Shandong, China; 4 Department of Anesthesiology, Geffen School of Medicine, University of California Los Angeles, Los Angeles, California, United States of America; Hokkaido University, Japan

## Abstract

Ghrelin is an orexigenic brain-gut hormone promoting feeding and regulating energy metabolism in human and rodents. An increasing number of studies have reported that ghrelin and its identified receptor, the growth hormone secretagogue receptor 1a (GHS-R1a), produces remarkably wide and complex functions and biological effects on specific populations of neurons in central nervous system. In this study, we sought to explore the in vivo effects of acute ghrelin exposure on lateral amygdala (LA) neurons at the physiological and behavioral levels. *In vivo* extracellular single-unit recordings showed that ghrelin with the concentration of several nanomolars (nM) stimulated spontaneous firing of the LA neurons, an effect that was dose-dependent and could be blocked by co-application of a GHS-R1a antagonist D-Lys3-GHRP-6. We also found that D-Lys3-GHRP-6 inhibited spontaneous firing of the LA neurons in a dose-dependent manner, revealing that tonic GHS-R1a activity contributes to orchestrate the basal activity of the LA neurons. Behaviorally, we found that microinfusion of ghrelin (12 ng) into LA before training interfered with the acquisition of conditioned taste aversion (CTA) as tested at 24 h after conditioning. Pre-treatment with either purified IgG against GHS-R1a or GHS-R1a antagonist blocked ghrelin’s effect on CTA memory acquisition. Ghrelin (12 ng) had no effect on CTA memory consolidation or the expression of acquired CTA memory; neither did it affect the total liquid consumption of tested rats. Altogether, our data indicated that ghrelin locally infused into LA blocks acquisition of CTA and its modulation effects on neuronal firing may be involved in this process.

## Introduction

Ghrelin is an octanoylated, 28-amino acid orexigenic peptide which is synthesized both peripherally in stomach and centrally in hypothalamus [1], [2]. So far, ghrelin is the only identified, perhaps the only existing natural ligand of growth hormone secretagogue receptor-1a (GHS-R1a), a highly conserved G-protein-coupled receptor (GPCR) with seven transmembrane domains [1]. Although there is highest expression level of GHS-R1a in the pituitary and hypothalamus highlighting its importance in GH release, food intake, body-weight regulation and energy homeostasis [1], [3], [4], [5], abundant GHS-R1a expression was also found in extra-hypothalamic regions, including cortex, hippocampus, ventral tegmental area (VTA) and many others [4], [6], [7], [8]. The broader distribution of GHS-R1a in central nervous systems (CNS) suggests that ghrelin/GHS-R1a signaling has important physiological functions beyond feeding control and energy metabolism. Indeed, increasing evidence has shown that ghrelin affects multiple higher CNS activities, including reward, mood, learning and memory.

It is well known that the amygdala is one of the key brain structures implicated in acquisition and storage of multiple types of aversive and emotional memory, including auditory fear conditioning and conditioned taste aversion [9], [10], [11], [12]. The amygdala consists of several anatomically and functionally distinct nuclei, including the lateral (LA) and basal (BA) nuclei (together referred to as the basolateral complex of amygdala) and the central nucleus (CeA). Since the LA nuclei receives multimodal sensory input from the thalamus and cortex, it is considered to serve as the major sensory interface. Many studies support the notion that the LA is an essential site where early, NMDA receptor-dependent synaptic plasticity is required for the acquisition of conditioned fear [9], [10], [13]. Projections and terminals of ghrelin neurons were identified in amygdala by means of immunohistochemistry [14]. Noticeably, a very recently study showed that in rat amygdala, GHS-R1a mRNA was most abundant in the lateral nucleus rather than the medial nucleus of amygdala [15]. Those evidences suggested that ghrelin/GHS-R1a signaling may modulate neuronal activity in basolateral complex of amygdala and thus affect aversive memory processes. Indeed, a very recent study showed that injection of ghrelin into the basolateral amygdala enhanced passive avoidance learning [16].

CTA is a very robust and widely used model for the study of aversive memory processes in which an animal learns to associate novel taste with visceral malaise. Like fear conditioning, CTA has also been used to identify the molecular, cellular, circuit and system mechanisms of acquisition, consolidation and extinction of memory. Previous studies showed that the basolateral complex of amygdala is one of the major parts of the neural circuits that subserve CTA [12], [17], [18]. Although several studies have shown that intra-amygdala injection of ghrelin enhances memory retention on a passive avoidance task [16], [19], [20], [21], so far there is no study demonstrating the possible effect of ghrelin on CTA memory formation. In addition, ghrelin was previously shown to directly increase the firing rate of NPY/AgRP neurons, GHRH neurons in the arcuate nucleus and dopaminergic neurons in the substantia nigra pars compacta as well [22], [23], [24], however its effect on neuronal excitability in lateral amygdala and the behavioral relevance has not been reported yet. Therefore, the aim of the present study is to explore the acute effects of ghrelin on neuronal activity within the LA and extend these findings to behavioral outputs of amygdala: CTA memory acquisition and expression.

## Materials and Methods

### Animals

Adult male Wistar rats (300–350 g) used in the experiments were purchased from the Experimental Animal Center at Lukang Pharmaceutical Co (Jining, China). All animals were housed individually in plastic Nalgene cages and maintained on a 12 hr light/dark cycle under controlled temperature (20–22°C). Standard rodent chow and water were available *ad libitum* except during each experimental session. Animals were allowed for acclimation in the colony room for two weeks before the start of any experiments. Both this study and the animal protocols used in this study were approved by the Chancellor’s Animal Research Committee at the Medical College of Qingdao University, in accordance with National Institutes of Health guidelines.

### Electrophysiological Recordings and Micro-pressure Ejection

Rats were anesthetized with 20% urethane (1 g/kg, i.p). Additional supplemental doses were administered intraperitoneally when necessary. A rectal temperature was maintained at 37–38°C using a heat control unit and heating pad. The rat was mounted in a stereotaxic device (Narishige, Tokyo, Japan). Burr holes were drilled in the skull to expose the cortex area overlying the LA (2.8 mm posterior to bregma, 5.2 mm lateral to the midline and 7.5–8.5 mm below the skull surface). After removal of the dura, warm agar (3% in saline) was applied to cover the open part of the brain surface. Triple-barrel microelectrode was slowly lowered into the lateral amygdala via a micromanipulator (MO-8; Narishige). Electrophysiological recordings and micro-pressure ejection were done as described previously [25].

Triple-barrel microelectrodes were constructed using a vertical microelectrode puller (Cat −51217, Stoelting, USA) and the tip was broken back under microscopic control. The electrode impedance measured *in vivo* was 15–20 MΩ with the whole tip diameter of 3–10 uM. The recording barrel of the electrode was filled with 2% pontamine sky blue in 0.5 M sodium acetate, the other two micro-pressure ejection barrels was filled with various drug solutions and connected to a 4-channel nano-liter pressure microinjector (CFT-8401, Medrich, China). Drugs were ejected onto the surface of firing cells with short pulse gas pressure (1500 ms, 5.0–15.0 psi).

Once the microelectrode was advanced into the LA, the extracellular action potential of single unit was isolated and recorded (with a signal-to-noise ratio of ≥3∶1 and a minimal duration of 1.0 ms). The recorded electrical signals were amplified by a micro-electrode amplifier (MEZ-8201, Nihon Kohden, Tokyo, Japan) and displayed on a memory oscilloscope (VC-11, Nihon Kohden, Tokyo Japan). Spike data acquisition and analysis were preprocessed with spike-2 software (Cambridge Electronic Design, UK). Stable baseline firing rate were obtained for at least 2 min before drug administration. After drug ejection, neuronal activity was continuously recorded for a minimum of 4 min before a subsequent administration occurred. Recording sites were marked by ejection of Pontamine sky blue at the completion of experiment.

### Brain Slice Preparation and Whole-cell Current Clamp Recordings

Brain slices were prepared as described previously ([12], [26]. Briefly, brains were rapidly removed from 17- to 20-days old wistar rats and placed in ice-cold artificial cerebral spinal fluid (ACSF) containing (in mM) 120 NaCl, 3.5 KCl, 20 NaHCO3, 10 D-glucose, 1.3 MgSO4, 2.5 CaCl2, and 1.25 NaHPO4. Coronal slices (350 mm thick) containing the amygdala were cut on a vibratome (Leica VT1000S). Slices were allowed to recover in oxygenated (95% O2/5% CO2) ACSF for 1 h at room temperature before experiments were performed. Slices were then transferred to the recording chamber and were continuously perfused with oxygenated ACSF at a rate of ∼2 ml min^−1^ at 31°C.

Cells were visualized with an upright microscope with infrared differential interference contrast (IR/DIC) technique (Olympus, Japan). Whole-cell current-clamp recordings were made from neurons in the LA with a Multiclamp 700B amplifier (Molecular Devices, Union City, CA). Electrodes (3–6 MΩ) contained (in mM) 120 potassium methylsulfate, 20 KCl, 10 HEPES, 0.2 EGTA, 2.0 MgCl2, 2 Mg_2_ATP, 0.3 Na_3_GTP, 7 phosphocreatine (pH 7.2–7.4, 290–300 mOsm). Responses were filtered at 2 kHz and digitized at 10 kHz. All data were acquired, stored and analyzed using pClamp10.0 (Molecular Devices). Access resistance and input resistance were monitored throughout the experiment. Only cells that reached the minimal criteria for health and stability (resting membrane potential more negative than −55 mV and access resistance less than 25 MΩ) were included in the analyses of this study.

LA neurons were identified on the basis of their action potential half-width and spike frequency adaptation in response to a long (600 ms duration) depolarizing current injection, as described previously [26]. To investigate the firing properties of LA neurons and their response to ghrelin, we delivered 15 current injection steps (600 ms duration) from −200 to 500 pA in 50 pA increments before and after the application of ghrelin. The recorded lateral amygdala cells were further divided into two groups on the basis of spike-frequency adaptation [26]. Rapidly adapting (RA) cells fired only 1–5 spikes in response to increasing amplitudes of current injections. Slowly adapting (SA) cells fired more than six action potentials during the current injections. Ghrelin (100 nM) were bath applied by adding it to the superfusate and were washed out by continuous perfusion with ACSF.

### Surgery and Microinfusion

Rats were implanted bilaterally with 22-gauge stainless steel cannulae into the LA under 8% chloral hydrate anesthesias (400 mg/kg, i.p). The stereotactic coordinates of LA nuclei complex were −2.8 mm anteroposterior, ±5.2 mm mediolateral and −7.5 mm dorsoventral relative to bregma, according to Paxinos and Watson (1998). The cannulae were anchored to the skull with stainless steel screws and dental cement. A 28-gauge dummy cannula was inserted into each cannula to prevent clogging. Rats were given analgesic and antibiotics injection immediately after surgery. Antibiotics treatment was continued for three days after surgery. Animals were given at least 5 d to recover before being subjected to experimental manipulations. For microinfusion, the dummy cannula was removed from the guide cannula and a 28-gauge infusion cannula, extending 0.8 mm from the tip of the guide cannula, was inserted. The infusion cannula was connected via PE20 tubing to Hamilton microsyringe driven by a microinfusion pump (Stoelting Co., USA). Microinfusion was performed bilaterally over 5 min. The infusion cannula was left in position before withdrawal for an additional 5 min to minimize dragging of the injected liquid along the injection tract.

Chemicals or vehicles were locally infused into lateral amygdala. Ghrelin, GHS-R1a antagonist D-Lys3-GHRP-6 and YIL781 were purchased from Tocris Bioscience (Minneapolis, MN, USA), purified IgG against GHS-R1a and control IgG were purchased from Phoenix Pharmaceuticals, Inc (Burlingame, CA, USA). The NMDA receptor antagonist, DL-2-amino-5-phosphonopentanoic acid (AP-5) and the AMPA receptor antagonist, 6-cyano-7-nitroquinoxaline-2, 3-dione (CNQX) were from Sigma (St Louis, MO, USA). The drugs were dissolved in vehicle (physiological saline or DMSO as indicated) and adjusted to pH 7.4. The particular drug dosages used for microinfusion as well as the timing of drug administration relative to training or retrieval, were selected on the basis of previous studies or preliminary experiments.

### Behavioral Procedure

After water deprivation for 24 h by removal of water bottle and change to new bedding, the thirsty rats were habituated over 5 days to obtain their daily water supply within 20 min from two serological pipettes, one containing 5 ml of tap water and the other containing the same volume of 50 mM NaCl. The rats were then assigned to groups with matched total water intake and body weight on the last day of habituation.

On training day, the rats were first presented with tap water and 50 mM NaCl pipettes for 10 min, then 150 mM LiCl (unconditioned stimuli, US) or 150 mM NaCl for 10 min. Twenty-four hour later, a multiple-choice test was performed to determine the acquired aversion to salty water (conditioned stimuli, CS). The rats were presented with an array of six pipettes for 20 min, three containing 50 mM NaCl and three containing tap water. The aversion index (AI) was defined as a percentage of water consumption in ml, 100X (water consumed)/total (water +NaCl)%. Hence, 50% is equal-preference. The higher AI means the better CTA memory.

### Histology

The cannula tip locations and recording electrode sites were finally confirmed by crystal violet staining at the end of the experiment. Rats were killed by overdose with anesthetic, decapitated, and the brains were removed and fixed in 10% formalin for a minimum of 24 hr. Brains were cryoprotected with 30% sucrose in 0.1 M phosphate buffer and were then frozen and sliced with a cryostat into 40 µm coronal sections. Mounted sections were then stained with cresyl violet. Only those animals with bilateral placements in the basolateral complex of amygdala were included in analysis. Recording sites were identified by the blue spot caused by ejection of pontamine sky blue.

### Data Analysis

Data was expressed as mean ± SEM unless indicated. Student’s *t* tests or ANOVA analysis followed by Dunnett’s test or Newman-Keuls multiple comparison test was used as required to examine the statistical significance of the drug effects. To analyze the in vivo electrophysiology data, the basal firing rate was determined by the averaged frequency of 120 s immediately before drug administration. The maximal change of firing frequency within 50 s following drug application was considered as drug effect. Drug effect was then expressed as the percentage of basal firing activity (%). For individual neuron, a change of at least 20% of basal firing rate during drug application was considered significant according to previous publication [25]. The duration of the action potentials recorded from LA units was quantified as the time from the initial change from baseline to the return to baseline. As for slice studies, the spike amplitudes were measured from resting potential. Action potential half-widths were measured as the spike width at the half-maximal voltage. Input resistance was calibrated by fitting the I–V curve with a linear regression. Neurons were identified as lateral amygdala neurons on the basis of their location and electrophysiological features [26].

## Results

### Ghrelin Dose-dependently Increases the Spontaneous Firing Rate of the LA Neurons

**Figure 1:**
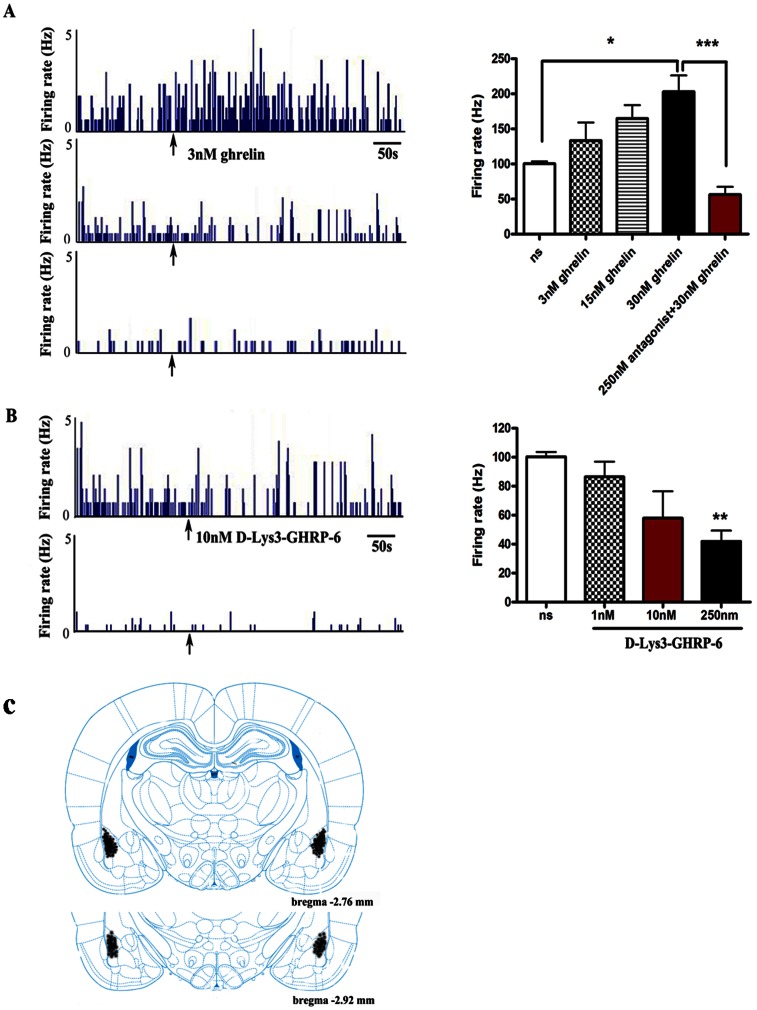
Ghrelin increases spontaneous firing rate of the LA neurons. **A**, Ghrelin increases spontaneous firing rate of the LA neurons in average, which is reversed by GHS-R1a antagonist D-Lys3-GHRP-6. **Left**, example firing rate histograms recorded in LA neurons that display increased, decreased or unchanged spontaneous firing after pressure microinjection of ghrelin at a concentration of 3 nM. **Right,** summarized data showing that ghrelin administration increases the spontaneous firing rate of LA neurons in a dose-dependent manner. Co-ejection of GHS-R1a antagonist D-Lys3-GHRP-6 (250 nM) with ghrelin (30 nM) not only reversed the effects of ghrelin, but further altered the firing rate beyond baseline levels. *n* = 8 neurons from 2 rats for saline control group, *n* = 16–20 neurons from 6 rats for ghrelin-treated groups. **B,** GHS-R1a antagonist D-Lys3-GHRP-6 reduces the basal firing rate of the LA neurons in a concentration-dependent manner. **Left**, example firing rate histograms recorded in two LA neurons with different basal firing rate, both displaying reduced firing after administration of 10 nM D-Lys3-GHRP-6. **Right**, summarized data showing that D-Lys3-GHRP-6 administration attenuated the frequency of spontaneous firing of LA neurons in a dose-dependent manner. *n* = 8 neurons from 2 rats for saline control group, *n* = 4–20 neurons from total 10 rats for D-Lys3-GHRP-6 treatment groups. Arrows indicate time of drug administration. *p<0.05, **p<0.01 or ***p<0.001 means significant, error bars indicate SEM. **C,** Illustration of reconstructed recording sites. Bilateral recording placements are indicated as closed circles in the basolateral amygdala complex, mainly in LA (n = 125 neurons). At brain structure diagrams the numbers refer to anterior-posterior distance from bregma in millimeter adapted from the stereotaxic atlas of Paxinos and Watson.

Consistent with the previous report [27], the majority of spontaneously spiking LA neurons showed relative low basal firing rate under our recording conditions (Median, 0.95 Hz; range, 0.03–19.03 Hz; *n* = 125 from 26 rats). Frequency distribution analysis showed that about 70% out of total 125 units recorded had a basal firing rate lower than 1 Hz. The broader basal firing range varying from 0.03 Hz to 19.03 Hz suggested that those units may belong to different neuronal subpopulations with distinct physiological and functional properties. Supportively, those spontaneously spiking neurons had variable responses to ghrelin challenge (Fig. 1A): thirty-two of 56 neurons (from 12 rats) displayed increased firing rate, while other 24 neurons showed no response to ghrelin or even decrease in firing rate after ghrelin application. There was no significant correlation (p>0.05) between basal firing rate and response to ghrelin, suggested that the ghrelin responsive neurons could not be differentiated based on their baseline firing rate.

Despite of the response variability, our statistical analysis revealed that ghrelin increased the frequency of spontaneous firing of LA neurons as shown in Fig. 1A, in a dose-dependent manner (one-way ANOVA, p<0.05; four groups, *n* = 8 for saline; *n* = 20, 16, 20 for 3 nM, 15 nM and 30 nM ghrelin respectively). In particular, the averaged firing rate of the LA neurons was changed from basal 1.17±0.24 Hz to 1.84±0.34 Hz by 30 nM ghrelin ejection. Dunnett’s multiple comparison test showed that this concentration of ghrelin significantly increased the spontaneous firing rate of LA neurons (203.1%±22.96% of basal firing rate, *n* = 20 neurons from 6 rats, compared to the saline group, p<0.05). Ejection of lower concentration of ghrelin (3 nM and 15 nM) also showed the increasing tendency on firing frequency. Saline ejection onto the surface of firing cells had no substantial effect on neuronal firing rate (100.40%±3.26% of basal firing rate, *n* = 8 from 2 rats), indicating that the firing rate changes observed after ghrelin application is not caused by pressure ejection itself. Moreover, ghrelin had no effect on the duration of the action potentials.

Next, we checked whether administration of GHSR1a antagonist, D-Lys3-GHRP-6, could abolish ghrelin’s effect on spontaneous fire rate of LA neurons. Clearly, co-ejection of 250 nM D-Lys3-GHRP-6 with 30 nM ghrelin not only reversed the effects of ghrelin (unpaired *t*-test, p<0.001), but further altered the firing rate beyond baseline levels (56.27%±11.17% of baseline, *n* = 20 from 6 rats, Fig. 1A). This phenomenon could attribute to a combination of two effects played by D-Lys3-GHRP-6 on LA neurons: (1) removal of ghrelin-induced actions and (2) attenuation of GHS-R1a tone on the same neuron. Hence, our data indicated that ghrelin directly facilitates neuronal firing in the LA through activation of GHS-R1a receptor.

### D-Lys3-GHRP-6 dose-dependently Inhibits Spontaneous Firing of the LA Neurons

To further check the effect of D-Lys3-GHRP-6 on spontaneous firing of LA neurons, we applied different concentration of D-Lys3-GHRP-6 (1 nM, 10 nM and 250 nM) to total 34 neurons from 10 rats. Twenty-nine of 34 neurons displayed decreases in firing rate after D-Lys3-GHRP-6 administration, while the other 2 neurons showed the opposite responses and the remaining 3 neurons had no response to D-Lys3-GHRP-6. In average, D-Lys3-GHRP-6 administration attenuated the frequency of spontaneous firing of LA neurons in a dose-dependent manner (one-way ANOVA, p<0.05, Fig. 1B). The averaged firing rate of the LA neurons was changed from 1.05±0.55 Hz to 0.97±0.59 Hz (*n* = 4), 1.44±0.49 Hz to 0.84±0.30 Hz (*n* = 10) and 1.27±0.40 Hz to 0.80±0.31 Hz (*n* = 20) after administration of 1, 10 or 250 nM D-Lys3-GHRP-6, respectively. Statistical analysis showed that the inhibition of 250 nM D-Lys3-GHRP-6 on firing rate was significant (41.91%±7.42% of pre-drug baseline values, *n* = 20, p<0.01 compared to the saline group, Dunnett’s test). Actually, lower concentration of D-Lys3-GHRP-6 (10 nM) produced a similar effect on spontaneous firing of LA neurons (58.0%±18.47% of baseline, *n* = 10), suggesting that the suppression of D-Lys3-GHRP-6 was close to be saturated at the concentration of 10 nM. Thus, our results indicated that tonic GHS-R1a activity contributes to maintain the basal activity of the LA neurons. Indeed, previous study showed that endogenous ghrelin signal through GHS-R1a is constitutively active [28].

### Ghrelin Increases the Number of Firing of LA Neurons in vitro

To better understand the firing properties of subtypes of neurons that respond to ghrelin, we performed whole-cell current clamp recordings in acute amygdala slices. A total of 31 LA neurons from 7 rats were recorded with the average resting membrance potential of −64±2.1 mV and input resistance of 142±24 MΩ. All neurons displayed wide action potential with half width of 1.32±0.10 ms, indicating that they were more likely projection neurons in LA [26]. Indeed, most of those neurons recorded showed varying degrees of spike frequency adaptation in response to a 600 ms depolarization current injection (Figure S1), which was consistent with the previous report [26]. Specifically, 20 out of 31 neurons recorded (65%) fired only one to five spikes in response to increasing amplitudes of 600 ms depolarizing currents (RA cells), whereas the remaining 11 cells (35%) fired between 6 and 30 action potentials during the current injections (SA cells). After bath application of 100 nM ghrelin for 6–8 min [29], sixteen out of total 31 neurons showed obvious increase in the number of action potentials elicited by depolarizing current injections, while the remaining 15 cells had no clear response to ghrelin (Figure S2). One cell with the resting potential of −57 mV even displayed spontaneous firing after ghrelin application. The effect of ghrelin could be partially or completely washed out after ACSF perfusion for ∼15 min. More interestingly, we found that among the 16 neurons excited by 100 nM ghrelin, 9 cells belonged to SA neurons which fired more than 6 spikes in response to 600 ms depolarizing current injections. Thus, our data suggested that the SA neurons (9/11) more likely be activated by ghrelin compared to RA cells (7/20). However, a larger number of neurons need to be investigated before drawing a clear conclusion. Since none of the 31 neurons could be clearly identified as an interneuron, we did not know whether and how ghrelin affects the activity of interneurons in the LA.

### The Effect of Intra-LA Infusion of AP-5 and CNQX on CTA Memory Processes

**Figure 2:**
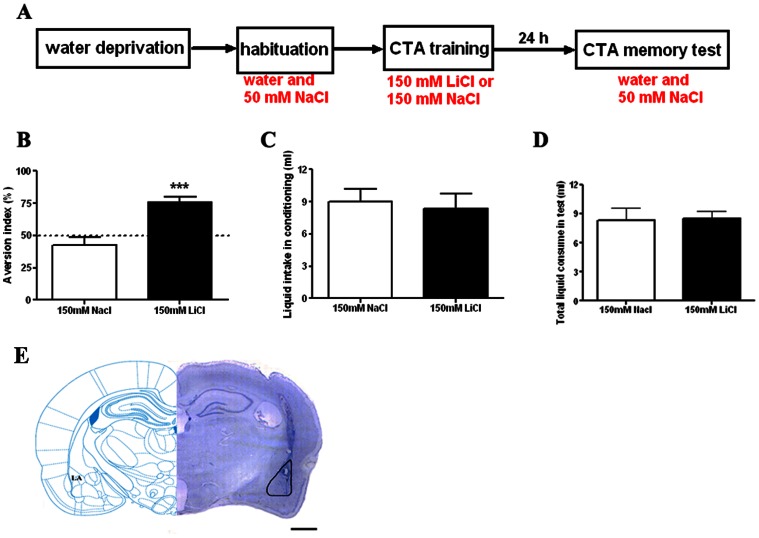
CTA paradigm used in the experiment. **A**, Illustration of the experimental procedures to generate CTA in rats. **B**, Summarized bar graph showing that rats acquires long-term taste aversion (24 h) to salty solution after drinking of LiCl, but not the NaCl. The LiCl group rats shows significantly higher aversion index (AI) than the control NaCl group during memory test, meaning good CTA memory. **C**, Summarized bar graph showing that the total liquid intake during conditioning was similar in LiCl and the control NaCl groups. **D**, A column graph showing that the total liquid consumption during test was similar in LiCl and the control NaCl groups. **E**, Representative photomicrographs illustrating placement of cannula and needle tip in the lateral amygdala. Line bar indicates 1 mm. ***p<0.001, *n* = 8 per group. Error bars indicate SEM.

In our CTA training paradigm, the lithium chloride solution (150 mM LiCl in drinking water) served as both the US and the CS because it induces nausea response after drinking, and its salty taste makes the animal acquiring taste aversion to another similar salty solution, for example 50 mM NaCl (Fig. 2A). Our result showed that 24 h after consumption of 150 mM LiCl but not the same amount of 150 mM NaCl (Fig. 2C), the thirsty rat preferred to drink more tap water than the test solution (50 mM NaCl, Fig. 2B). Importantly, the total liquid consumption during test was similar in LiCl and NaCl trained groups (p>0.05, Fig. 2D). Statistical analysis indicated that the AI values of the two groups were significantly different (75.84%±4.21% for LiCl group and 42.71%±6.22% for NaCl group, *n* = 8 per group, *t*-test, p<0.001, Fig. 2B). Thus, those results indicated that our CTA model was adoptable to the following experiments.

**Figure 3:**
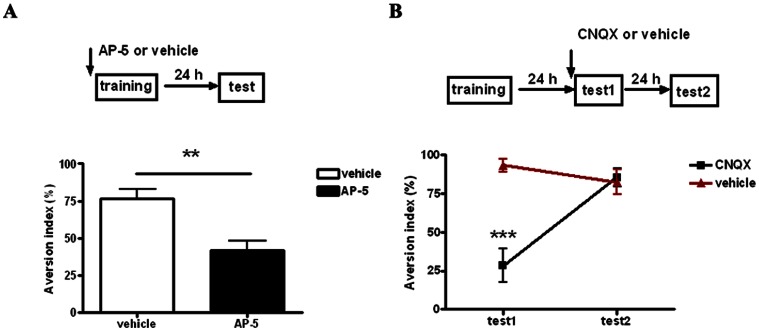
The effect of intra-LA infusion of AP-5 and CNQX on acquisition and expression of CTA memory. **A**, Illustrating the effect of AP-5 on CTA memory acquisition. **Top**, schematic of the experimental design. **Bottom**, intra-LA infusion of AP-5 (5 µg/0.5 µl per side) before training blocks the acquisition of CTA memory tested at 24 h after conditioning. *n* = 7 for AP-5 group and *n* = 8 for vehicle group. **B**, Illustrating the effect of CNQX on CTA memory expression. **Top**, intra-LA administration of CNQX (1 µg/0.5 µl per side) before test1 blocks CTA memory expression. The blockade is reversible since intact CTA memory can be recalled 24 h later during test2. *n* = 7 for CNQX group and *n* = 5 for vehicle group. **p<0.01 or ***p<0.001 means significant. Error bars indicate SEM.

Previous study showed that visceral aversive stimuli and gustatory information can be processed in parallel on several levels including the NTS, amygdala, insular cortex, and etc [30]. To confirm that the LA is an important structure where CS-US associations are essential to form CTA, we first checked the effect of AP-5 administration on aversive memory acquisition. As shown in Fig. 3A, intra-LA infusion of AP-5 (5 µg/0.5 µl per side) 20 min before training blocked the acquisition of CTA memory as tested at 24 h after conditioning. The 24 h AI value of the AP-5 group was approximate to random (50%) and significantly smaller than that of the vehicle group (unpaired *t*-test, p<0.01, *n* = 7 for AP-5 group and *n* = 8 for vehicle group). We also checked the effect of CNQX on aversive memory expression. Administration of CNQX (1 µg/0.5 µl per side) twenty minutes before memory recall blocked CTA memory expression as shown by small AI compared to vehicle group (unpaired *t*-test, p<0.001, *n* = 7 for CNQX group and *n* = 5 for vehicle group, Fig. 3B). The effect of CNQX on memory expression was transient; the CTA memory could be fully retrieved 24 h later as shown in Fig. 3B. To restrict the action of glutamatergic blockade in LA and to minimize involvement of the basolateral amygdala [31], we repeated the above experiments with the reduced volume of CNQX and AP-5. As shown in Figure S3, local infusion of 0.2 µl CNQX shortly before test blocked CTA memory expression while 0.3 µl AP-5 had no effect on memory retrieval. Altogether, our results demonstrated that NMDA receptor activation and AMPA receptor activation in the LA are required for CTA acquisition and expression respectively. Hence, we confirmed here that the LA is an important structure for CTA learning and memory processing.

### Intra-LA Infusion of Ghrelin Blocks Acquisition of CTA, but had no Effect on Memory Consolidation or Expression

**Figure 4:**
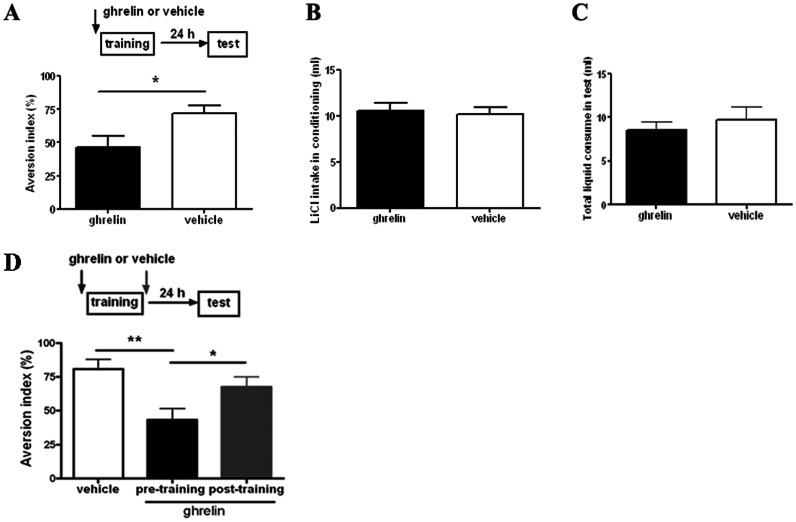
Microinfusion of ghrelin into the LA interferes with the CTA memory acquisition, but has no effect on memory consolidation. **A**, Illustrating of ghrelin’s effect on CTA acquisition.**Top**, schematic of the experimental design. **Bottom**, intra-LA infusion of ghrelin (12 ng, 0.75 µl per side) before training impairs acquisition of CTA, represented by significant smaller AI tested at 24 h after conditioning, compared to the vehicle group. **B**, Showing that the two groups of animals intakes similar amount of LiCl during conditioning. **C**, Showing that the two groups of animals consumes similar amount of liquid during tests. *n* = 20 for each group. **D**, Comparison of ghrelin’s effect on CTA memory consolidation versus acquisition.**Top**, schematic of the experimental design. Same dose of ghrelin was infused with reduced volume compared to that applied in **A**. **Bottom**, intra-LA infusion of ghrelin (12 ng, 0.5 µl per side) shortly after conditioning has no effect on CTA memory. *n* = 9–10 for each group. *p<0.05 and **p<0.01 means significant. Error bars indicate SEM.

Since both ghrelin and its receptor GHS-R1a were expressed in the LA, and more importantly, ghrelin was shown to modulate the spontaneous firing of LA neurons, we were interested to check if ghrelin affects the acquisition and expression of CTA in system level. Interestingly, we found that microinfusion of ghrelin (12 ng, 0.75 µl per side) into the LA 20 min before training interfered with the acquisition of CTA memory. As shown in Fig. 4A, ghrelin treatment group displayed significant smaller AI compared to the vehicle group, as tested at 24 h (p<0.05) after conditioning (unpaired *t*-test, *n* = 20 for each group). Importantly, the ghrelin-treated animals and vehicle-treated animals drank identical volume of LiCl solution during conditioning (Fig. 4B) and presented similar nausea response as well. Also, the two groups of animals consumed similar amount of liquid during tests (p>0.05, Fig. 4C). Microinfusion of same dose of ghrelin with reduced volume (12 ng, 0.5 µl) into the LA produced similar blockade on CTA memory acquisition (p<0.01 compared to vehicle group, n = 9 or 10, one-way ANOVA followed by Newman-Keuls multiple comparison test, Fig. 4D). To check the possible effect of ghrelin on memory consolidation, we infused same dose of ghrelin (12 ng, 0.5 µl) into the LA 20 min after CTA training in a separated group of animals. Our data indicated that, different from the effect of pre-training infusion, post-training infusion of ghrelin did not significantly impaired CTA memory formation in tested rats (Fig. 4D). Therefore, our results demonstrated here that intra-LA infusion of nanograms of ghrelin blocked acquisition, but not consolidation of CTA memory.

**Figure 5:**
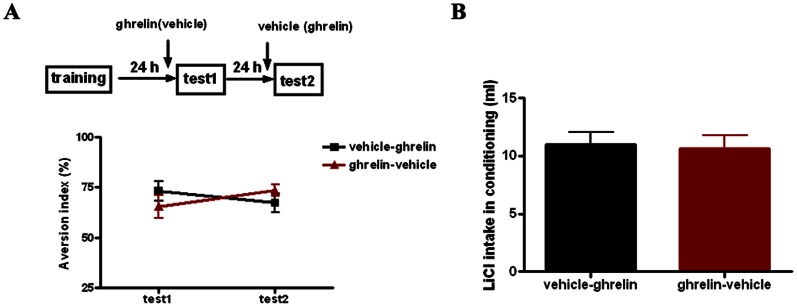
Microinfusion of ghrelin into the LA has no effect on CTA memory expression. **A**, Illustrating of ghrelin’s effect on CTA memory expression.**Top**, schematic of the experimental design. **Bottom**, intra-LA infusion of ghrelin (12 ng, 0.5 µl per side) shortly before test has no effect on memory retrieval, represented by similar AI showing in test 1 and test2. **B**, Showing that the two groups of animals intake similar amount of LiCl during conditioning. *n* = 16 for each group. Error bars indicate SEM.

To check the possible effect of ghrelin on memory expression, we first trained animals without drug infusion, then divided them into two groups with comparable taste aversion performance. One group of animals was thus treated with ghrelin 20 min before test1 and vehicle 20 min before the following test2 in 24 h interval. The other group was treated in a reversed order (Fig. 5A). Our results showed that the two groups of animals displayed comparable AI in both tests, indicating that ghrelin had no effect on memory retrieval (n = 16 per group, one-way ANOVA, p>0.05, Fig. 5).

**Figure 6:**
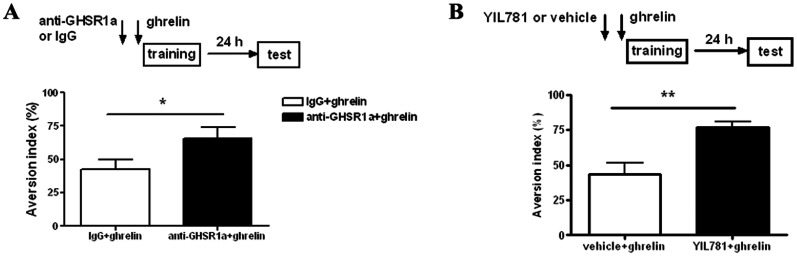
Ghrelin’s effect on memory acquisition is abolished by pre-administration of IgG against GHS-R1a or selective GHS-R1a antagonist. **A**, Pre-administration of IgG against GHS-R1a reverses ghrelin’s effect on CTA memory acquisition. **Top**, schematic of the experimental design. **Bottom,** summarized results showing that the anti-GHS-R1a IgG (0.5 mg/ml, 0.75 µl per side) pretreated group presents significantly higher AI than the control group. *n* = 16 for each group. **B**, Pre-administration of GHS-R1a antagonist, YIL781, erases ghrelin’s effect on CTA memory acquisition. **Top**, schematic of the experimental design. **Bottom,** summarized results showing that YIL781 (750 µM, 0.5 µl per side) pretreated group presents significantly higher AI than the control group. *n* = 8 for each group. *p<0.05 or **p<0.01 means significant. Error bars indicate SEM.

Finally, to check if ghrelin’s effect on memory acquisition is mediated by activation of GHS-R1a, we pre-treated the LA neurons with purified rabbit IgG against GHS-R1a (0.5 mg/ml, 0.75 µl per side) or selective GHS-R1a antagonist YIL781 (750 µM, 0.5 µl per side) 20 min before local infusion of ghrelin during training. The anti-GHS-R1a IgG pre-treated group showed significantly higher AI than the IgG control group as tested 24 h later (p<0.05, *n* = 16 for each group, unpaired *t*-test, Fig. 6A). More dramatically, pre-treated with YIL781 completely erased the blockade of ghrelin on CTA memory acquisition (p<0.01, *n* = 8 for each group, unpaired *t*-test, Fig. 6B). These results indicated that pretreatment with either anti-GHS-R1a IgG or GHS-R1a antagonist reversed ghrelin’s effect on CTA acquisition. Altogether, our present data demonstrated that intra-LA infusion of ghrelin blocked CTA acquisition, which was mediated by GHS-R1a activation.

## Discussion

In this study, we demonstrated that local infusion of neuropeptide ghrelin into the LA blocks CTA acquisition. The effect seems to be specific based on the following findings: (1) Ghrelin had no acute effect on drinking since the total liquid consumption during training or test are comparable between ghrelin and vehicle treated groups; (2) Ghrelin had no effect on US processing since ghrelin-treated rats showed similar nausea response as the controls; (3) During training, ghrelin-treated animals drink similar volume of 150 mM LiCl as the controls; (4) Administration of ghrelin shortly after conditioning has no effect on CTA memory consolidation. (5) Application of the same dose of ghrelin had no effect on expression or recall of CTA memory. (6) Ghrelin displayed the same effect when the intra-LA infusion volume was reduced to 0.5 µl per side. All those findings suggested that ghrelin blocks CTA memory acquisition by interruption of CS-US association in LA by certain mechanisms.

A large number of behavioral studies in rodents have suggested that ghrelin promotes learning and memory. For example, previous studies showed that systemic, intracerebroventricular or intra-nucleus (hippocampus, amygdala and dorsal raphe nucleus) injection of ghrelin dose-dependently enhanced memory retention in an avoidance task in both rats [19], [20], [32] and mice [33]. Of note, this effect could be observed only when ghrelin was injected before training, but not before retrieval and only for long term memory [34]. Novel object recognition was also enhanced by ghrelin administration to the hippocampus in rats [20]. Subcutaneous injections of ghrelin or the ghrelin mimetic LY444711 led to a marked improvement in spatial memory retention in mice [33]. Moreover, ghrelin receptor deficient mice expressed impairments in spatial learning [35] and exogenous ghrelin rescued deficits shown by ghrelin−/− mice in a novel object recognition test [33]. However, in this study we provided certain evidence showing that ghrelin and GHS-R1a signaling have opposing effects on learning and memory, at least for CTA memory. Our result is not completely surprising. Memory impairments after ghrelin administration had actually been reported in neonatal chicks [36]. A very recent study further showed that GHS-R1a knockout mice exhibited clearly better performance in both Morris water maze and open field, suggesting that GHS-R1a activation actually interferes with acquisition of spatial memory. Supportively, preliminary studies in our laboratory are also showing that intra-CA1 administration of ghrelin blocks spatial memory formation (unpublished observations). Interestingly, in contrast to its memory enhancing effects in rodents, serum ghrelin levels were recently shown to be negatively correlated with declarative memory in elderly adults [37]. Also, the observed effects of ghrelin on procedural memory consolidation seemed to be impairments rather than promotions [38].

To define the site where ghrelin takes action, we slowly infused 12 ng ghrelin into the basolateral complex of amygdala (mainly in the LA) instead of systemic or icv injection used by previous studies [19], [32], [33], [39]. Noticeably, we administrated a rather smaller amount of ghrelin compared to others [15], [16], [20], [40]. Not only affected learning and memory, previous studies demonstrated that intra-amygdala injection of relatively high dose of ghrelin simultaneously caused complicated, controversial effects on emotional response, including anxiety, depression, feeding, and etc [15], [16], [20], [21], [40], which could have big impact on cognition. Note that, in our studies, same amount of ghrelin blocking CTA memory did not change the total liquid intake as shown in both training and the test phase of our CTA paradigm, neither did it affect anxiety as test in open filed and elevated plus maze (data not shown). Therefore, we predicted that the suppressive effect of ghrelin on CTA memory acquisition is more likely attributed to interruption of CS-US association in the LA rather than changes of anxiety or locomotor activity induced by ghrelin administration. It should be noted, however, that more precise study may be necessary to confirm that the interruption of CS-US association caused by ghrelin infusion is specific in the LA. Considering that ghrelin receptor deficient mice was reported to display normal passive avoidance learning compared to wild types [35], it would also be interesting to test if this mutant mice showed excellences or deficits on CTA memory. Besides different brain regions and drug dose usage, experimental procedure, sensitivity of the behavioral paradigms, genetic background and age of animals may also account for some of the discrepancies among different studies. Altogether, our study provided further evidence that central ghrelin and GHS-R1a signaling has diverse actions on higher brain functions including learning and memory, which is brain region specific and probably dependent upon metabolic status [6].

Although many behavioral studies showed that ghrelin and GHS-R1a signaling are involved in learning and memory, the precise molecular and cellular mechanisms mediating the modulation effects of ghrelin on memory processes are still uncertain. Previous study showed in mice that peripheral injections of ghrelin increased dendritic spine formation in CA1 regions of hippocampus, while ghrelin-knockout mice have reduced spine density compared to controls [33]. Furthermore, ghrelin promotes the generation of long term potentiation (LTP) in both mice and rats [33], [41]. Since all those studies were done in hippocampus and relevant to spatial memory, no evidence so far showing whether or not the similar plasticity changes happened in amygdala and relevant to CTA acquisition. Besides learning-induced modifications of the synaptic strength, studies have revealed that changes in neuronal excitability might also serve as mechanisms through which a neural circuit is set to a permissive state to facilitate synaptic modifications that are necessary for memory storage [42]. Supportively, aversion conditioning was reported to result in neuronal activity changes in response to a CS taste in both the BLA and insular cortex, which was maintained throughout memory test session [43], [44], [45]. Moreover, several studies showed that ghrelin directly increased the firing rate of NPY/AgRP neurons, GHRH neurons and dopaminergic neurons [22], [23], [24]. Therefore we explored the effects of ghrelin on neuronal activity within the LA considering its possible relevance to CTA memory encoding.

Our results showed that ghrelin dose-dependently increased the overall excitability of LA neurons, an effect that could be totally abolished by GHS-R1a antagonist. Moreover, GHS-R1a antagonist lowered the basal firing of LA neurons in a dose-dependent manner, indicating that GHS-R1a, active constitutively [28] or by circulating ghrelin, may contribute to fine-tuning of the basal activity of those neurons. Despite the reported positive correlations between excitability and memory, previous studies also demonstrated that the global increase in excitability actually caused learning and memory deficits by reducing flexibility of the neuronal networks involved [42], [46], [47], [48], [49]. Moreover, considering that in principle only a small portion of eligible neurons in a network participates in encoding a given memory, global increases in network excitability during conditioning may interfere with memory allocation, thus impairs memory [12], [50], [51]. Therefore, it seems reasonable to propose that the increase in neuronal excitability evoked by ghrelin exposure to the LA might disrupt CTA memory acquisition through certain network mechanisms.

The lateral structures of amygdala consist of heterogeneous subpopulations of neurons with distinct function, in which different subtypes of neurons may respond differently to ghrelin. Supportively, our in vivo recordings showed that LA neurons displayed diverse response to ghrelin, suggesting that they might belong to different subpopulations. To identify the subtype of LA neurons responsive to ghrelin, we performed whole-cell current clamp recordings in acute amygdala slices. Our results showed that bath application of 100 nM ghrelin increased the number of firing in 16 out of 31 projection neurons in LA. More interestingly, we found that the slowly adapting neurons (SA neurons) were more likely excited by ghrelin than the rapidly adapting ones (RA neurons). However, we should be aware that about 15% neurons in the lateral and basolateral amygdala are local GABAergic interneurons [52], ghrelin may directly enhance the excitability of those interneurons leading to increased network inhibition as well [24]. Unfortunately, the present study did not address the possible effect of ghrelin on interneurons in the LA. Thus, further studies are needed to fully clarify the cell-type specific actions of ghrelin in the lateral amygdala, which will help us better understand the relationships between the cellular and behavioral effects of ghrelin. In addition, very recently, ghrelin was reported to reduce the frequency of mEPSCs recorded from large pyramidal-like neurons in LA [15]. Altogether, our studies and results from other laboratories suggested that ghrelin, by acting on GHS-R1a, modulates neuronal excitability and synaptic transmission, which may work together to interfere with CTA memory acquisition.

There is still a lack of knowledge concerning the precise molecular mechanisms and transmitters involved mediating ghrelin’s effect on neuronal activity and learning. GHS-R1a is well-known as a GPCR that is linked primarily to G_α11/q_–phospholipase C (PLC) signaling pathways, leading to hydrolysis of PtdIns(4,5)*P*2 (PIP2) and production of IP3 and DAG as a result [3]. The actions of the G_q_ G-protein activated by GHS-R1a/ghrelin may also couple with the reduction in M-channel through hydrolysis of PIP2 by PLC. M-channel is a PIP2-regulated non-inactivating potassium current which is important in raising the threshold for firing an action potential [53]. It is unique because it is open at rest and even more likely to be open during depolarization. Furthermore, when the muscarinic acetylcholine receptor is activated, the channel closes. Besides the M channel, PIP_2_ also regulate the function of other ion channels, including Na^+^ channels and inwardly rectifying potassium channels (Kir channels), which also play important roles in regulating cell membrane excitability [54]. Besides G_11/q_ G-protein, ghrelin/GHS-R1a is also linked to G_αi/o_ signaling pathways [55], [56]. Other signaling pathways involved are ERK1/2, Raf-MEK-MAPK, PKA, PKC, PI3K/Akt/GSK3β etc [57]. It seems that although GHS-R1a generates intracellular signaling mainly through its G_α11/q_ subunit, the specific intracellular pathways elicited by this receptor seem to be dependent on the tissue type in which it is expressed [41], [58], [59], [60]. As for the transmitters involved, one possibility is that ghrelin may act as a modulator of other neurotransmitters such as glutamate, dopamine or GABA. Of particular relevance, D1 receptor antagonist blocked the effects of ghrelin on the object location memory task [61], while GHS-R1a is required for DRD2-induced feeding suppression in mice [62]. It is thus possible that GHS-R1a may modulate dopamine signaling in vivo on learning and memory.

In conclusion, our study further proved that ghrelin and GHS-R1a take diverse actions in the brain besides regulation of energy metabolism. Before proposals about cognition enhancing drugs targeting the central ghrelin receptor are warrantable, much more research is needed to elucidate the effects and underlying mechanisms of ghrelin and its receptor agonists on memory, especially on human cognition.

## Supporting Information

Figure S1 **Representation of the three different firing patterns of LA projection neurons. A–C**, Three LA cells that exemplify the varying response to a large current injection (600 ms, 400 pA). Neuron A (RA neuron) fires less than 5 spikes, while neuron B (SA neuron) fires more than 6 spikes, neuron C fires even more and shows no apparent spike adaptation during 600 ms current injection. All the three neurons have resting membrane potentials around −62 mV.
(TIF)

Figure S2 **Representation of the LA neurons with different responses to 100 nM ghrelin.** **A,** Sample LA neuron showing increase in the number of action potentials after ghrelin administration. **B,** Sample LA neuron showing no response to ghrelin. **A–B**, **Top traces**, basal neuronal firing elicited by depolarizing current injections when perfusion with ACSF. **Bottom traces**, neuronal firing elicited by same depolarizing current injections when bath perfusion with 100 nM ghrelin. **C,** A series of depolarizing current injections (600 ms duration) applied to neuron A and B in order to evoke action potentials.
(TIF)

Figure S3 **The effect of intra-LA infusion of reduced volume of AP-5 or CNQX on expression of CTA memory.** **A**, Illustrating the effect of CNQX (0.2 µl) on CTA memory expression. **Top**, schematic of the experimental design. **Bottom**, intra-LA infusion of CNQX (0.5 µg/0.2 µl per side) before test2 blocks the expression of CTA memory. **B**, Illustrating the effect of AP-5 (0.3 µl) on CTA memory expression. **Top**, schematic of the experimental design. **Bottom**, intra-LA administration of AP-5 (3 µg/0.3 µl per side) before test2 does not block memory expression. *n* = 7 for each group. ***p<0.001 means significant. Error bars indicate SEM.
(TIF)
