# Supplementary figures and images for: Ghrelin Modulates Lateral Amygdala Neuronal Firing and Blocks Acquisition for Conditioned Taste Aversion
Source: PLoS One. 2013 Jun 7;8(6):e65422. doi: 10.1371/journal.pone.0065422 (PMC3676403; doi:10.1371/journal.pone.0065422)

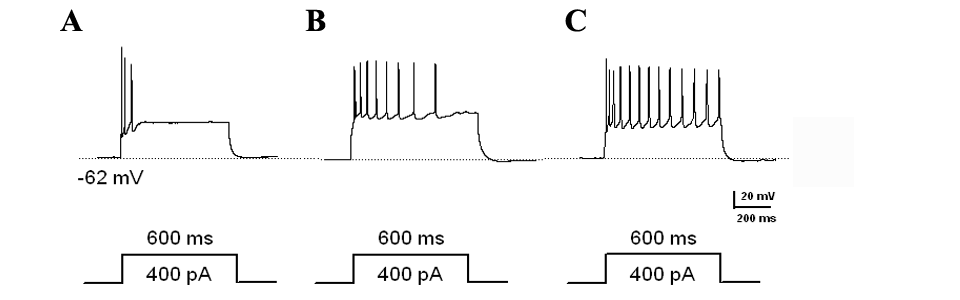

Supplement: Figure S1 — Representation of the three different firing patterns of LA projection neurons. A–C, Three LA cells that exemplify the varying response to a large current injection (600 ms, 400 pA). Neuron A (RA neuron) fires less than 5 spikes, while neuron B (SA neuron) fires more than 6 spikes, neuron C fires even more and shows no apparent spike adaptation during 600 ms current injection. All the three neurons have resting membrane potentials around −62 mV. (TIF) [file pone.0065422.s001.tif]

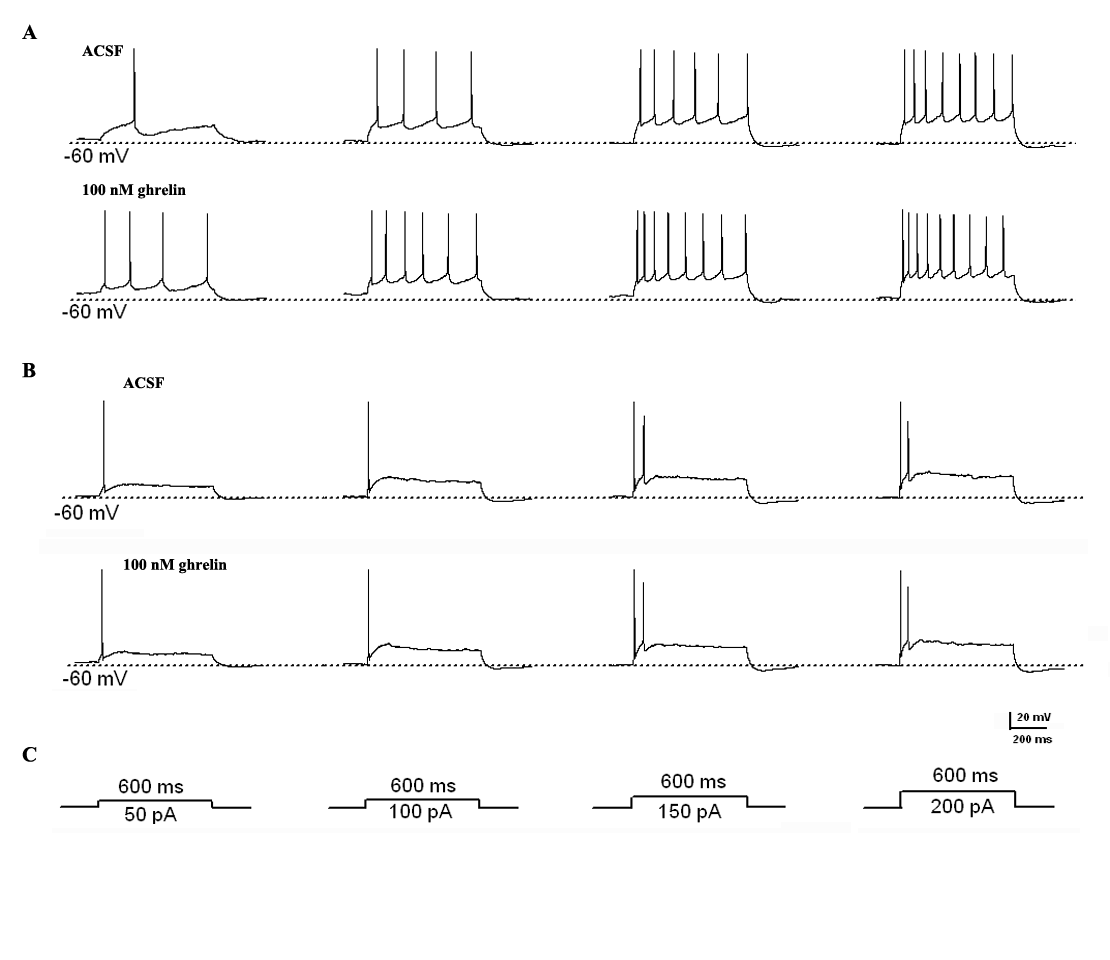

Supplement: Figure S2 — Representation of the LA neurons with different responses to 100 nM ghrelin. A, Sample LA neuron showing increase in the number of action potentials after ghrelin administration. B, Sample LA neuron showing no response to ghrelin. A–B, Top traces, basal neuronal firing elicited by depolarizing current injections when perfusion with ACSF. Bottom traces, neuronal firing elicited by same depolarizing current injections when bath perfusion with 100 nM ghrelin. C, A series of depolarizing current injections (600 ms duration) applied to neuron A and B in order to evoke action potentials. (TIF) [file pone.0065422.s002.tif]

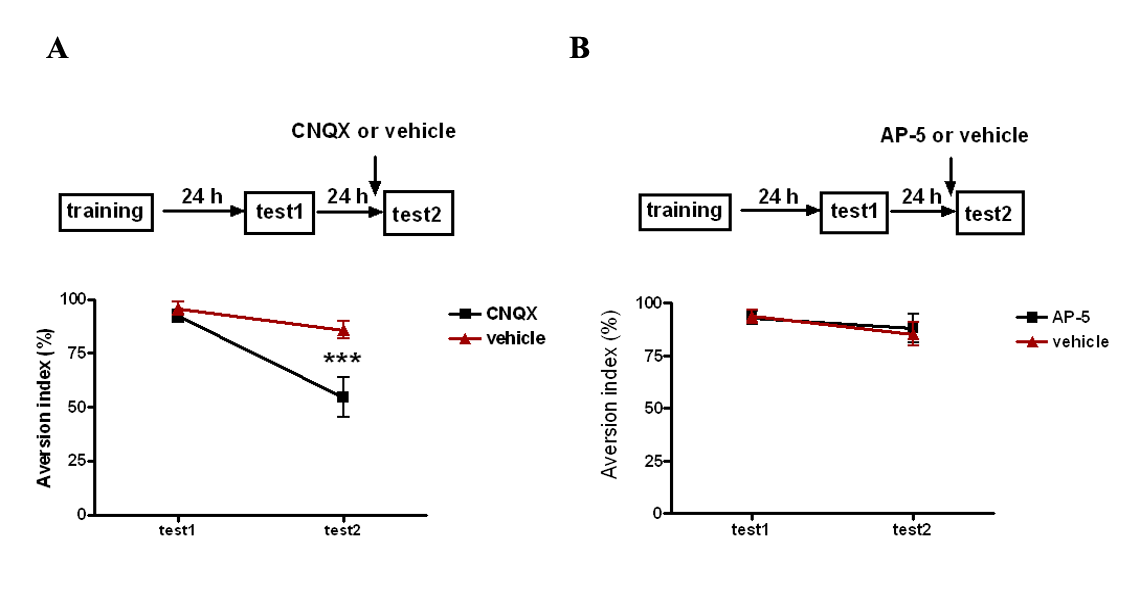

Supplement: Figure S3 — The effect of intra-LA infusion of reduced volume of AP-5 or CNQX on expression of CTA memory. A, Illustrating the effect of CNQX (0.2 µl) on CTA memory expression. Top, schematic of the experimental design. Bottom, intra-LA infusion of CNQX (0.5 µg/0.2 µl per side) before test2 blocks the expression of CTA memory. B, Illustrating the effect of AP-5 (0.3 µl) on CTA memory expression. Top, schematic of the experimental design. Bottom, intra-LA administration of AP-5 (3 µg/0.3 µl per side) before test2 does not block memory expression. n = 7 for each group. ***p<0.001 means significant. Error bars indicate SEM. (TIF) [file pone.0065422.s003.tif]
